# Chemerin Is an Antimicrobial Agent in Human Epidermis

**DOI:** 10.1371/journal.pone.0058709

**Published:** 2013-03-20

**Authors:** Magdalena Banas, Katarzyna Zabieglo, Gopinath Kasetty, Monika Kapinska-Mrowiecka, Julia Borowczyk, Justyna Drukala, Krzysztof Murzyn, Brian A. Zabel, Eugene C. Butcher, Jens M. Schroeder, Artur Schmidtchen, Joanna Cichy

**Affiliations:** 1 Department of Immunology, Faculty of Biochemistry, Biophysics and Biotechnology, Jagiellonian University, Kraków, Poland; 2 Department of Cell Biology, Faculty of Biochemistry, Biophysics and Biotechnology, Jagiellonian University, Kraków, Poland; 3 Department of Computational Biophysics and Bioinformatics, Faculty of Biochemistry, Biophysics and Biotechnology, Jagiellonian University, Kraków, Poland; 4 Division of Dermatology and Venerology, Department of Clinical Sciences, Lund University, Lund, Sweden; 5 Department of Dermatology, Zeromski Hospital, Kraków, Poland; 6 Palo Alto Institute for Research and Education, Veterans Affairs Palo Alto Health Care System, Palo Alto, California, United States of America; 7 Stanford University School of Medicine, Stanford, California, United States of America; 8 Department of Dermatology, University Hospital Schleswig-Holstein, Kiel, Germany; Charité, Campus Benjamin Franklin, Germany

## Abstract

Chemerin, a chemoattractant ligand for chemokine-like receptor 1 (CMKLR1) is predicted to share similar tertiary structure with antibacterial cathelicidins. Recombinant chemerin has antimicrobial activity. Here we show that endogenous chemerin is abundant in human epidermis, and that inhibition of bacteria growth by exudates from organ cultures of primary human skin keratinocytes is largely chemerin-dependent. Using a panel of overlapping chemerin-derived synthetic peptides, we demonstrate that the antibacterial activity of chemerin is primarily mediated by Val^66^-Pro^85^, which causes direct bacterial lysis. Therefore, chemerin is an antimicrobial agent in human skin.

## Introduction

Chemerin is a multifunctional protein implicated in chemotaxis of immune cells, regulation of differentiation and metabolic function of adipocytes, and glucose homeostasis [1], [2], [3]. It binds with high affinity to three receptors, chemokine-like receptor 1 (CMKLR1) and atypical chemokine CC motif receptor-like 2 (CCRL2) as well as G protein-coupled receptor 1 (GPR1). However, among these receptors, only CMKLR1 is responsible for direct chemerin-mediated chemotactic effects [4], [5]. Chemerin mRNA is present in many tissues, including liver, fat, placenta, pancreas, lung and skin [6], [7]. Chemerin is also present in plasma in the nanomolar range. Like other serum proteins, the liver may be a primary source for circulating blood chemerin [3]. However, chemerin is also expressed by epithelial cells, including kertinocytes [8], although the biological significance of chemerin in skin remains unknown.

Human chemerin is secreted as a 143-amino acid precursor, pro-chem163S. Proteolytic processing of the C-terminus of pro-chem163S is required for this protein to become an active chemoattractant. Chemerin lacking 6 amino acids from the C-terminus, thus ending at serine^157^ (chem157S), appears to be the most effective form in controlling chemotaxis of several types of immune cells. Among cells responsive to chemerin gradients are plasmacytoid dendritic cells (pDCs), macrophages and NK cells [7], [9], [10], [11], [12]. Serine proteases of the inflammatory cascade, such as neutrophil elastase and cathepsin G, as well as host cysteine proteases including cathepsin L and K or pathogen-derived staphopain B, are potent activators of chemerin chemotactic activity [13], [14], [15]. These enzymes can process chemerin *in vitro* to generate bioactive chemerin isoforms identical to the endogenous isoforms isolated from body fluids [16]. However, extensive cleavage of this protein that has been reported to occur either *in vitro* or *in vivo*, also results in generating chemerin isoforms that lack chemotactic activity [3], [17], [18]. These data suggest that at least some chemerin fragments may play other, not chemotaxis-related functions.

Chemerin expression in the skin is not uniform and varies based on anatomic position as well as disease state. Chemotactically active chemerin was detected in lesional skin of psoriasis patients, where it may contribute to selective pDC recruitment [11], [19]. However, psoriatic lesions show much lower chemerin levels in the epidermis compared to the healthy skin, but strong chemerin immunoreactivity in the dermis. This is in contrast to normal skin where chemerin is primarily expressed by epidermal keratinocytes, but rarely, if at all, in the dermis [19], [20]. Therefore, chemerin reactivity in the epidermis suggests an additional, non-pDC-recruitment-related role for this protein in skin biology.

The predicted structural homology between chemerin and antimicrobial cathelicidins such as cathelin-like N-terminal region of human hCAP18 [6], [13], [21], [22], led us to hypothesize that chemerin may confer some protection against invading microbes. This was supported by our previous studies demonstrating antimicrobial activity of two chemerin isoforms (chemS157 and chemR125) against *E. coli* and *K. pneumoniae* [13]. These recombinant chemerin isoforms lack 6aa and 38 aa, and terminate at Ser^157^ and Arg^125^, respectively. Although both isoforms differed significantly in supporting chemotaxis, they were equally effective in reducing the growth of *E. coli* [13]. These data suggest that different chemerin domains are responsible for chemotactic and antimicrobial properties of this protein.

Since recombinant chemerin was previously used in order to demonstrate its antibacterial properties, it was important to determine whether chemerin exhibits antimicrobial functions in the skin environment, and whether its activity comprises a significant component of the secreted antibacterial products of skin. In this work we show that chemerin originating from exudates from organ cultures of human skin keratinocytes displays antimicrobial activity. Moreover, using chemically-synthesized chemerin-derived peptides we provide mechanistic information on the action of chemerin as well as insights into the domains that mediate its antimicrobial activity.

## Materials and Methods

### Peptides

Peptides were generated by a peptide synthesis platform (PEPscreen®, Custom Peptide Libraries, Sigma Genosys). MALDI-ToF Mass Spectrometry was performed on these peptides, and average Crude Purity of the peptides was found to be 60–70%. In addition, peptide 4 was synthesized and purified >98% by thinkpeptides, UK.

### Peptide selection

Mean hydrophobicity (H), and relative mean hydrophobic moments (rHM) were calculated using Combined Consensus Scale (CCS) of hydrophobicity [Tossi-2002] according to definitions given by Eisenberg et al. [23] with periodicity angle set to 100° and 160°, for α-helical and twisted β-strand conformations, respectively. All calculations were performed using an in-house program (hm.py). rHM by definition gives the value of mean hydrophobic moment relative to a perfectly amphipathic peptide of certain length, i.e. the amino acid sequence which maximizes rHM when adopting a given conformation. For CCS and 20 amino acid peptides, perfectly amphipathic peptides have the following sequence: RFFRRFFRRFRRFFRRFFRF (α-helix) and RFRFRRFRFRFRFRRFRFRF (twisted β strand).

### Cell culture

All human studies were performed in compliance with ethical protocols KBET/72/B/2008 and KBET/44/B/2011 approved by Jagiellonian University Institutional Bioethics Committee. Declaration of Helsinki protocols were followed. All participants provided their written informed consent to participate in these studies as recommended by the ethical board. Normal human keratinocytes were isolated from excess skin from donors obtained at the time of cosmetic surgery for mole removal or during plastic surgery. Skin biopsies were rinsed 3 times in calcium- and magnesium-free PBS supplemented with penicillin (5000 U/ml) – streptomycin (5 mg/ml) (all from Sigma). After washing, the biopsy was placed in PBS containing dispase (12 U/mL, Gibco) for 16 h in 4°C. Next, the epidermis was separated from the dermis with forceps followed by treatment with 0.05% trypsin with 2 mM EDTA (Sigma) to isolate epidermal cells. Cells were cultured in serum free KGM-Gold medium (Lonza Group Ltd.) to generate passage 1 cells. The keratinocytes were then plated at density of 5×10^4^ cells per well on permeable Transwell inserts (6.5-mm-diameter, 0.4 µm pore size; Falcon Transwell-Clear supports) in KGM-Gold medium. Cells were cultured at 37°C in presence of 5% CO_2_ until confluence. Polarized skin structures that resemble *in vivo* stratified epidermis were generated by air-liquid interface cultures for 1 to 3 weeks. Conditioned media were collected two days after the cells were exposed to the air-liquid interface and then every 48 h. The pulled conditioned media was analyzed.

### Preparation of epidermis lysate

The epidermis was separated from the dermis as described above. Epidermis was then homogenized in a RIPA buffer (25 mM Tris-HCl, pH 7.6, 150 mM NaCl, 1% NP-40, 1% sodium deoxycholate, 0.1% SDS) containing protease inhibitors (Complete, Roche), passed through a 40 µm cell strainer and incubated o/n at 4°C. Extracts were centrifuged at 10,000 g for 30 min to remove cellular debris and then normalized based on protein concentration as determined by BCA assay (Sigma). Lysates were stored at −20°C until used.

### Immunohistochemistry

Paraffin 6-µm sections were prepared from skin biopsies or keratinocyte cultures. Sections were blocked with goat IgG and stained with the rabbit anti-human chemerin (H-002-52 Phoenix Pharmaceuticals) or control IgG (normal rabbit IgG, Jackson Immunoresearch) followed by APC-goat anti-rabbit IgG F(ab)2 (Jackson Immunoresearch). The sections were counterstained with Hoechst 33258 (Invitrogen). Images were captured with a fluorescence microscope (NIKON, Eclipse) and analyzed by NIS elements software (Nikon).

### ELISA

Chemerin in conditioned media or in epidermis lysates was quantified by ELISA. Monoclonal mouse-anti-human chemerin (R&D System) Abs were used to capture chemerin and biotin-labeled polyclonal goat anti human chemerin (R&D System) followed by HRP-labeled streptavidin (BD PharMingen) were used to quantitate chemerin. The reaction was developed with TMB substrate (BD Science).

### Chemerin depletion

Chemerin was removed form keratinocyte conditioned media by immunoprecipitation with sepharose-conjugated anti-chemerin Abs. The conjugation of anti-chemerin IgG (G-002-52 rabbit anti-human chemerin, Phoenix Pharmaceuticals) or control IgG (normal rabbit IgG, Jackson Immunoresearch) to Sepharose4B (Pharmacia) was performed according to the manufacturer's recommendations.

### Microtitre broth dilution (MBD) assay

The antimicrobial activity of the indicated chemerin peptides or keratinocyte conditioned media against *Escherichia coli* (HB101, a conventional laboratory strain) was estimated as previously described [24]. Briefly, bacteria were grown in Mueller Hinton Broth (MHB) (Difco) to mid-logarithmic phase and used for subsequent experiments at 2–5×10^5^ or 2×10^4^ colony-forming units (CFU)/ml. Chemerin levels in the keratinocyte conditioned media did not exceed 20 ng/ml. Therefore, to investigate the antimicrobial effect of these media, we used 10 times less bacteria (2×10^4^ CFU/ml) compared to the standard MBD assay. Bacterial suspensions in MHB were mixed with diluent (90%:10%–10 mM HEPES or 50%:50% keratinocyte growth media) (control), chemerin peptides or keratinocyte conditioned media and incubated at 37°C for 18–24 h. After serial dilutions with MHB, the diluted mixture was plated on MHB agar plates and incubated at 37°C overnight for enumeration of CFU. In selected experiments, samples of the bacteria/peptide mixtures were also analyzed by spectrophotometry. These methods produced comparable results to the colony-forming assay (data not shown).

### Microdilution assay

Test microorganisms were incubated with various concentrations of chemerin-derived peptide 4 (>98% pure) in 10 mM sodium phosphate buffer pH 7.4 containing 1% (v/v) trypticase soy broth (TSB) for 2 h at 37°C. The antimicrobial activity was then analyzed by plating serial dilutions of the incubation mixtures and determining the number of CFU the following day.

### Radial diffusion assay

The indicated bacteria were grown to mid-logarithmic phase in 10 ml of full-strength (3% w/v) TSB as previously described [25]. The microorganisms were then washed once with 10 mM Tris, pH 7.4. 4×10^6^ bacterial CFUs were then added to 15 ml of the underlay agarose gel, consisting of 0.03% (w/v) TSB, 1% (w/v) low electroendosmosis type (EEO) agarose (Sigma) and 0.02% (v/v) Tween 20 (Sigma), with or without 0.15 M NaCl. The underlay was poured into a Ø 144 mm Petri dish. After agarose solidification, 4 mm-diameter wells were punched and 6 µl of chemerin-derived peptides or LL37 (Innovagen AB) was added to each well. Plates were incubated at 37°C for 3 hours to allow diffusion of the peptides. The underlay gel was then covered with 15 ml of molten overlay (6% TSB and 1% Low-EEO agarose in dH_2_O). Antimicrobial activity of a peptide was visualized as a zone of clearing around each well after 18–24 hours of incubation at 37°C.

### Lytic assay

*E. coli* JM83 strain containing plasmid pCH110 (Pharmacia-Amersham) encoding beta-galactosidase and ampicillin-resistance genes was grown in Luria-Bertani medium (LB) (Difco) containing 1.25 µg/ml ampicillin. All assays were performed using mid-logarithmic phase bacteria inoculated from overnight culture. Triton-lysed bacteria were served as 100% control. The pH-dependence was determined in 20 mM citrate-phosphate buffers of indicated pH (no NaCl), whereas the salt-dependence was assayed in 20 mM phosphate buffer with a constant pH = 7.2 containing the indicated amount of NaCl. To detect β-galactosidase activity, p-nitrophenyl-β-D-galactopyranoside, was used as a substrate.

## Results

**Figure 1:**
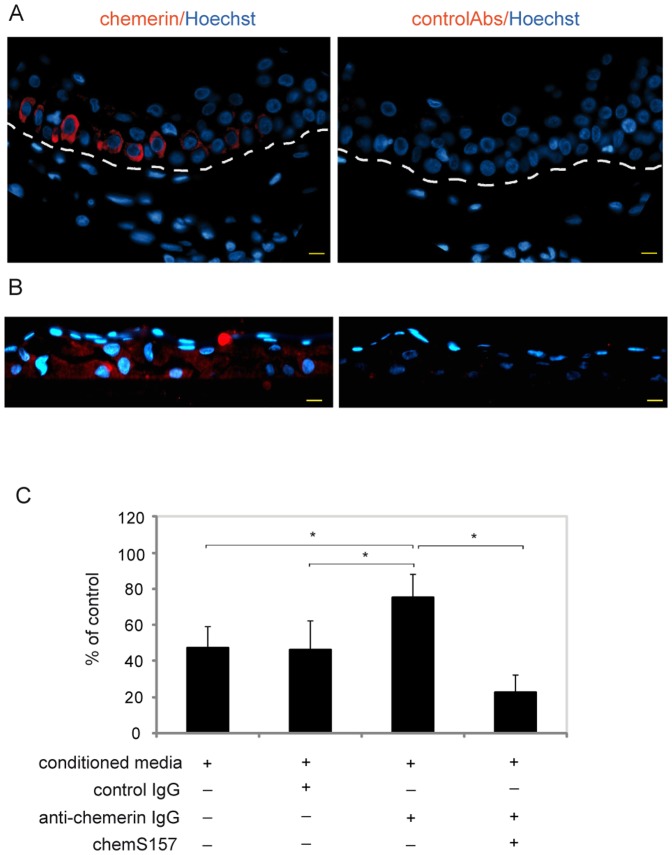
Keratinocyte-derived chemerin displays anti-bacterial activity. Paraffin sections of normal, shoulder skin biopsies (A) or chest keratinocytes grown in 3D culture for 1 week (B) were stained for chemerin or control rabbit Abs (red), with Hoechst counterstain to detect cell nuclei (blue). The slides were examined by fluoresce microscopy. Dotted lines in A indicate location of epidermis. Scale bar = 10 µm. Data are representative of three different donors. The antimicrobial activity of conditioned media from 3D cultures of keratinocytes (conditioned media) was tested against *E. coli* using the microtitre broth dilution assay (C). Where indicated, the conditioned media were first treated with sepharose-conjugated anti-chemerin Ab (anti-chemerin IgG), sepharose-conjugated control IgG (control IgG), or anti-chemerin Ab followed by recombinant chemerinS157 (chemS157) at 20 ng/ml. The results are expressed as the mean ± SD of four independent experiments. Statistically significant differences are indicated by asterisks (p≤0.01, Student's *t* test).

To investigate the role of chemerin in antibacterial defense of epithelial tissue, we first determined chemerin levels in lysates obtained from epidermis of healthy individuals. Since previous studies showed strong chemerin RNA expression in the epidermis from healthy individuals, which we confirmed by RT-QPCR (data not shown), we focused on quantifying chemerin protein in the skin. Indeed, chemerin protein was abundant in epidermis samples from multiple anatomic positions (824±424 ng per mg of total protein, n = 6) by ELISA (Table 1). Immunohistochemistry of paraffin embedded healthy skin derived from a shoulder biopsy revealed that chemerin is primarily expressed in the basal and suprabasal layers of epidermis, although its expression can also be detected in upper layers (Fig. 1A and data not shown). Based on the expression level and location of chemerin in healthy skin, chemerin is well-positioned to provide protection against skin-colonizing bacteria.

**Table 1:** Chemerin levels in lysates isolated from epidermis of healthy donors.

Patient number	Gender	Age	Anatomic location	Body mass index	Chemerin ng/mg of total protein
1	F	50	thigh	25.15	441.4
2	F	28	back	21.18	1293.2
3	F	26	nape of the neck	18.67	734
4	M	55	temple	27.15	417.8
5	M	57	the inside of elbow	26.51	1398.8
6	M	33	neck	22.09	655.5
**Mean ± SD**					**823±424**

Unlike standard cultures of normal human keratinocytes, tissue-like 3-dimensional structures express high levels of chemerin [20]. Therefore, to determine whether keratinocyte-derived chemerin is equipped with antimicrobial activity we established polarized cultures of keratinocytes isolated from healthy human skin derived from a chest biopsy. Interestingly, chemerin levels were the highest in the most matured 3D cultures, suggesting that differentiation status influence the chemerin expression (data not shown). *In vitro* cultured skin expressed chemerin in the basal- and suprabasal-like epithelial layers, correlating with its localization in situ in normal skin (Fig. 1B). To determine whether chemerin is a relevant antimicrobial agent in human keratinocytes, we tested conditioned media from these 3D cultures for antibacterial chemerin activity using MBD assays. We used *E. coli* for these studies, since human skin is frequently exposed to this bacteria. As demonstrated in Fig. 1C, the keratinocyte conditioned media (chemerin level ∼20 ng/ml) significantly inhibited the growth of *E. coli* strain H101, leading to survival of 47±12% of bacteria compared to vehicle-treated *E. coli* set as 100%. We previously used this strain to show inhibition of the bacterial growth by recombinant chemerin isoforms chemS157 and chemR125 [13]. To define the contribution of chemerin to the bacterial killing, we depleted chemerin from the conditioned media using sepharose-bound anti-chemerin Abs. Treatment of the supernatants with sepharose-bound anti-chemerin Abs reduced chemerin levels from 17–18 ng/ml to <10 pg/ml (below the limit of ELISA detection); sepharose-bound control Abs had no major effect on chemerin levels (not shown). The depletion of chemerin from the conditioned media significantly increased the survival of bacteria from 47±12% to 75±13%, whereas the conditioned media treated with sepharose-bound control Abs had no effect (Fig. 1C). Moreover, reconstitution of the conditioned media devoid of endogenous chemerin with recombinant human chemS157 (20 ng/ml) restored the killing activity of the conditioned media (bacterial viability significantly decreased to 22±10%) (Fig. 1C). Taken together, these data suggest that chemerin significantly contributes to the antibacterial properties of keratinocyte secretions.

**Figure 2:**
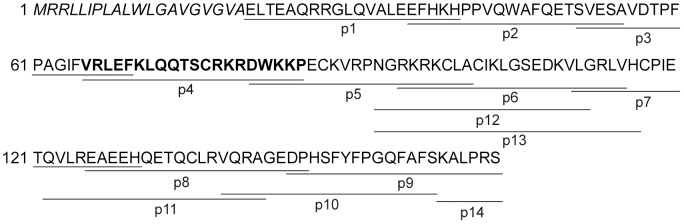
Overlapping peptides (p1-p14) are underlined in the chemerin sequence. The N-terminal signal peptide is indicated by italics. Peptide 4 is shown in bold.

To define the potential antimicrobial epitopes of chemerin, we selected and chemically synthesized 14 partially overlapping peptides covering the entire chemerin sequence (Fig. 2 and Table 2). These peptides, each ∼20 residue long, were selected to cover a wide range of net charge, mean hydrophobicity, and relative mean hydrophobic moment (rHM) values, allowing us to evaluate different determinants that might constitute the antibacterial activity of chemerin. The amphipathicity of chemerin peptides was analyzed by comparison of the rHM values, assuming for each of the peptide two distinct conformations: the α-helical and a β-structure. Owing to the presence of hydrophobicity patterns in native proteins [23], a substantially higher value of the calculated rHM for one of the alternative peptide structures (rHMα for α-helical and rHMβ for a β-structure) indicates the more probable conformation of the peptide. The analysis of the rHMβ/rHMα ratio for different reference peptides (Table 2 and data not shown), allowed us to classify p1–p14 peptides with the ratio>1.4 as showing propensity to adopt β-structures and those with the ratio<0.7 to adopt the α-helical conformation. Interestingly, all 20 residue long chemerin peptides with the net-charge higher than +2 clearly prefer a β-structure rather than the α-helical structure (Table 2), suggesting that the peptide conformation may be non-helical in the intact structure.

**Table 2:** Synthetic chemerin peptides^1^.

Name	sequence	H	rHMβ	rHMα	ratio	netchg
p1	ELTEAQRRGLQVALEEFHKH	−2.40	0.124	**0.466**	**0.27**	0.1
p2	EFHKHPPVQWAFQETSVESA	−1.58	0.169	0.204	0.83	−0.9
p3	SVESAVDTPFPAGIFVRLEF	0.42	**0.247**	0.109	**2.27**	−2.0
p4	**VRLEFKLQQTSCRKRDWKKP**	−3.28	**0.375**	0.157	**2.40**	5.0
p5	DWKKPECKVRPNGRKRKCLA	−4.41	**0.295**	0.205	**1.44**	6.0
p6	RKCLACIKLGSEDKVLGRLV	−1.27	**0.170**	0.119	**1.43**	3.0
p7	LGRLVHCPIETQVLREAEEH	−1.53	0.095	0.080	1.18	−0.9
p8	EAEEHQETQCLRVQRAGEDP	−4.44	0.244	0.177	1.38	−3.4
p9	DPHSFYFPGQFAFSKELPRS	−0.56	0.205	0.250	0.82	0.5
p10	VQRAGEDPHSFYFPGQFAFS	−0.59	0.223	0.186	1.20	−0.5
p11	QVLREAEEHQETQCLRVQRA	−3.58	0.141	**0.278**	**0.51**	−0.4
p12	NGRKRKCLACIKLGSEDKVL	−2.81	**0.284**	0.046	**6.20**	4.0
p13	NGRKRKCLACIKLGSEDKVLGRLVH	−2.34	0.168	0.167	1.00	5.5
p14	KALPRS	−2.63	0.100	**0.609**	**0.16**	2.0
pg-1	RGGRLCYCRRRFCVCVGR	−2.56	**0.376**	0.227	**1.66**	6.0
mag-2	GIGKFLHSAKKFGKAFVGEIMNS	−0.58	0.104	**0.505**	**0.21**	3.5

Peptide 4 is shown in bold. The net charge at pH 6 (netchg), mean hydrophobicity (H), relative mean hydrophobic moment assuming a β structure and α-helix, (rHMβ) and (rHMα) respectively, and rHMβ/rHMα ratio are indicated for each peptide. Data for the antibacterial peptide protegrin-1 (pg-1) and magainin-2 (Mag-2) known to adopt β structure and α-helical conformation, respectively when bound to the lipid membrane [36], [37], are shown for comparison. The rHM values and rHMβ/rHMα ratio for preferred peptide conformation are shown in bold.

1 chemerin peptides-patent pending.

**Figure 3:**
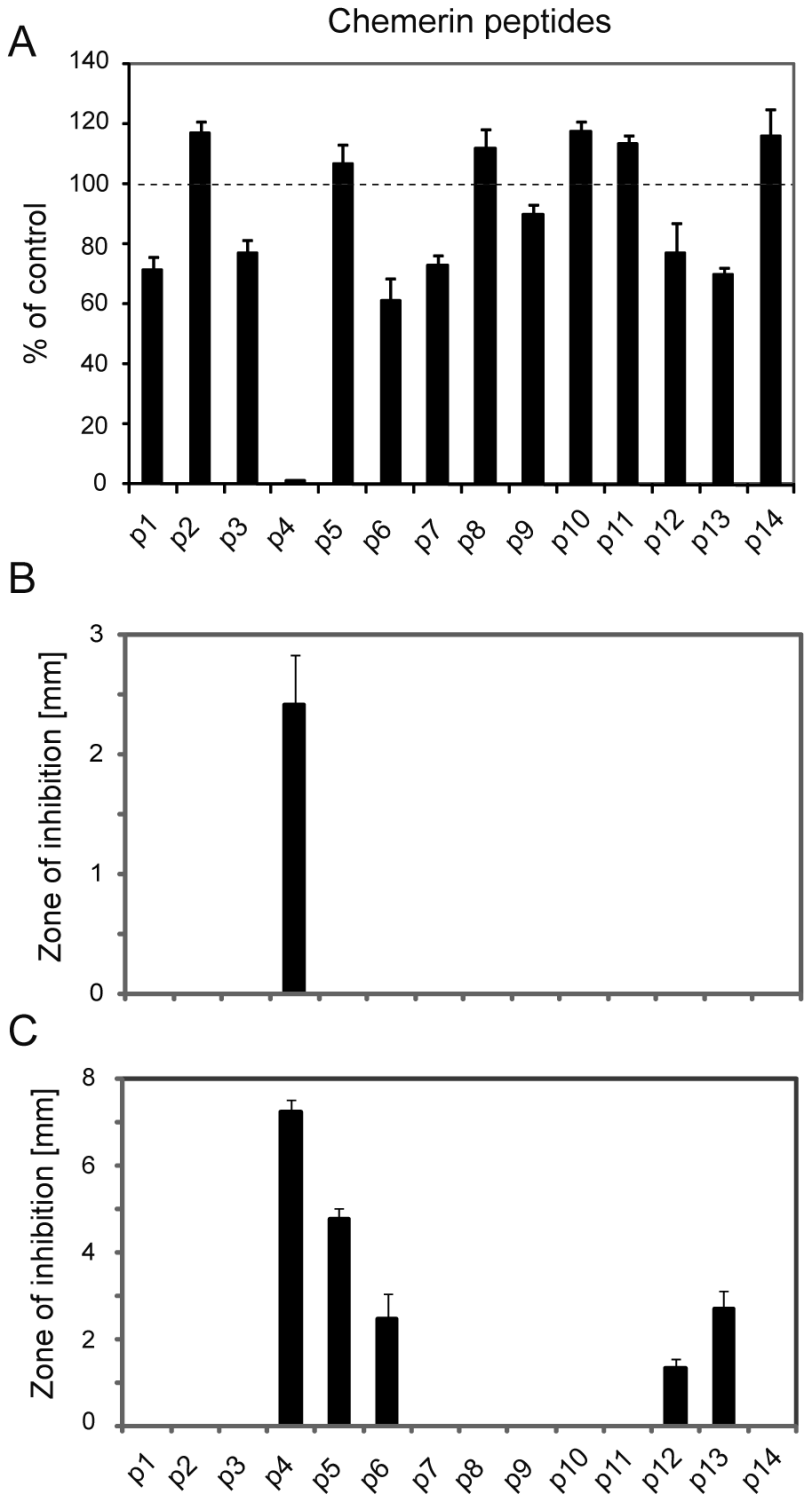
The chemerin-derived peptide 4 (Val^66^-Pro^85^) strongly inhibits growth of *E. coli*. Chemically synthesized chemerin peptides (p1-p14) were tested against *E. coli* HB101 using the microtitre broth dilution assay (A) or against *E. coli* ATCC 25922 using radial diffusion assay (RDA) in physiological 0.15 M NaCl (B) or low salt concentration (C). Bacteria were incubated with the peptides at 100 µM. The results are expressed as the mean ± SD of three independent experiments.

The selected chemerin-derived peptides (100 µM) were tested for antibacterial activity against *E. coli* strains HB101 and ATCC 25922 using the MBD and RDA assays, respectively. Several peptides inhibited growth of *E. coli* to some degree. Among them, peptide 4 (Fig. 2 and Table 2) corresponding to internal Val^66^-Pro^85^ region of human chemerin exhibited the strongest antimicrobial potency, resulting in almost complete inhibition of viable counts of *E. coli* H101 following 24 h treatment (Fig. 3A). P4 also significantly inhibited growth zones of *E. coli* ATCC 25922 in RDA under physiological salt conditions (0.15 M NaCl) (Fig. 3B). Other peptides such as p5 and p6 inhibited growth zones in low salt conditions, however, their inhibitory effects were less robust than p4 (Fig. 3C). For comparison, using a similar MBD assay, we previously demonstrated that pro-chem163S and chemS157 (evaluated at 2 µM) significantly inhibited bacterial growth, resulting in 59±13% and 33±15% *E. coli* survival, respectively [13]. Thus, the analysis of overlapping chemerin-derived peptides demonstrate that the region Val^66^-Pro^85^ of chemerin mediates the majority of the antibacterial activity of the full-length or chemotactically active chemerin, although cationic regions further C-terminal of p4 may also contribute to the resulting antibacterial activity of the intact holomolecule.

**Figure 4:**
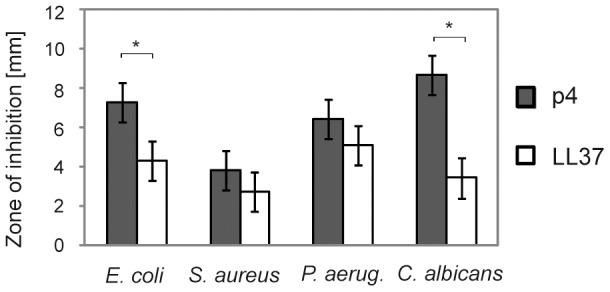
Chemerin peptide 4 exhibits anti-microbial activity against variety of microbial species. The indicated microorganisms (*E. coli* ATCC 25922, *S. aureus* ATCC 29213, *P. aeruginosa* ATCC 27853 and *C. albicans* ATCC 90028) were tested for antimicrobial activity of chemerin peptide 4 or LL-37 (both at 100 µM), using RDA assay. The results are expressed as the mean ± SD of three independent experiments. * p<0.005 (Student's *t* test).

We next examined a collection of clinically relevant human pathogens known to colonize the skin for sensitivity to peptide 4. The peptide was purified by HPLC to >98% and tested for antimicrobial activity using *E. coli* ATCC 25922, *S. aureus* ATCC 29213, *P. aeruginosa* ATCC 27853, as well as *C. albicans* ATCC 90028 by RDA assay. As demonstrated in Fig. 4, p4 at 100 µM inhibited the growth of all microorganisms, although it was particularly effective against Gram-negative bacteria, especially *E. coli*, but also the fungus *Candida*. Moreover, at 100 µM p4 was more potent in inhibiting growth of *E. coli* and *C. albicans* than the well-known keratinocyte-expressed antimicrobial agent LL-37 (Fig. 4). Similar results were generated with p4 against alternative strains of each and when p4 was tested at 40 µM (data not shown). The strong anti-microbial activity of p4 was further demonstrated by minimal inhibitory concentration (MIC) values which were in the range of 3.1–6.3 µg/ml (1.2–2.4 µM) for the most susceptible *E. coli*, to 12.5 µg/ml (4.8 µM) for the least susceptible *S. aureus* (Table 3). P4 also effectively inhibited the growth of two strains of *Staphylococcus epidermidis*, a common commensal skin bacteria (MIC = 12.5 µg/ml, Table 3). The MIC values were within the concentration range of most well-known anti-microbial agents [26]. Collectively, these data demonstrate that chemerin-derived peptide 4 is a potent anti-microbial agent.

**Table 3:** MIC values for indicated microorganisms as determined by microdilution assay.

p4 (µg/ml)	*E. coli* ATCC 11775	*S. aureus* ATCC 6538	*P. aerugin*. ATCC 10145	*C. albicans* ATCC 24433	*S. epiderm*. ATCC 12228	*S. epiderm*. ATCC 14990
100	100	100	100	100	100	100
50	100	100	100	100	100	100
25	100	100	100	100	100	100
12.5	100	100	100	100	100	100
6.3	100	98.9	100	100	99.7	99.1
3.1	100/98.6	72	99.4	80	96.8	97
1.6	92.1	57	96.7	39	83	84
0.8	82	23	71	18	61	38
0.4	57	11	23	7	16	34
0.2	16	0	6	14	17	17
0.1	20	0	17	0	8	8
0.05	7	0	0	0	0	0
0.02	26	0	0	0	0	0
0.01	0	0	0	0	0	0
**MIC (µg/ml)**	**3.1–6.3**	**12.5**	**6.3**	**6.3**	**12.5**	**12.5**

Data in columns indicate % of killing for each strain. The MIC was defined as the lowest concentration of p4 showing no visible growth (100% of killing). Mean of at least 3 measurements is shown.

**Figure 5:**
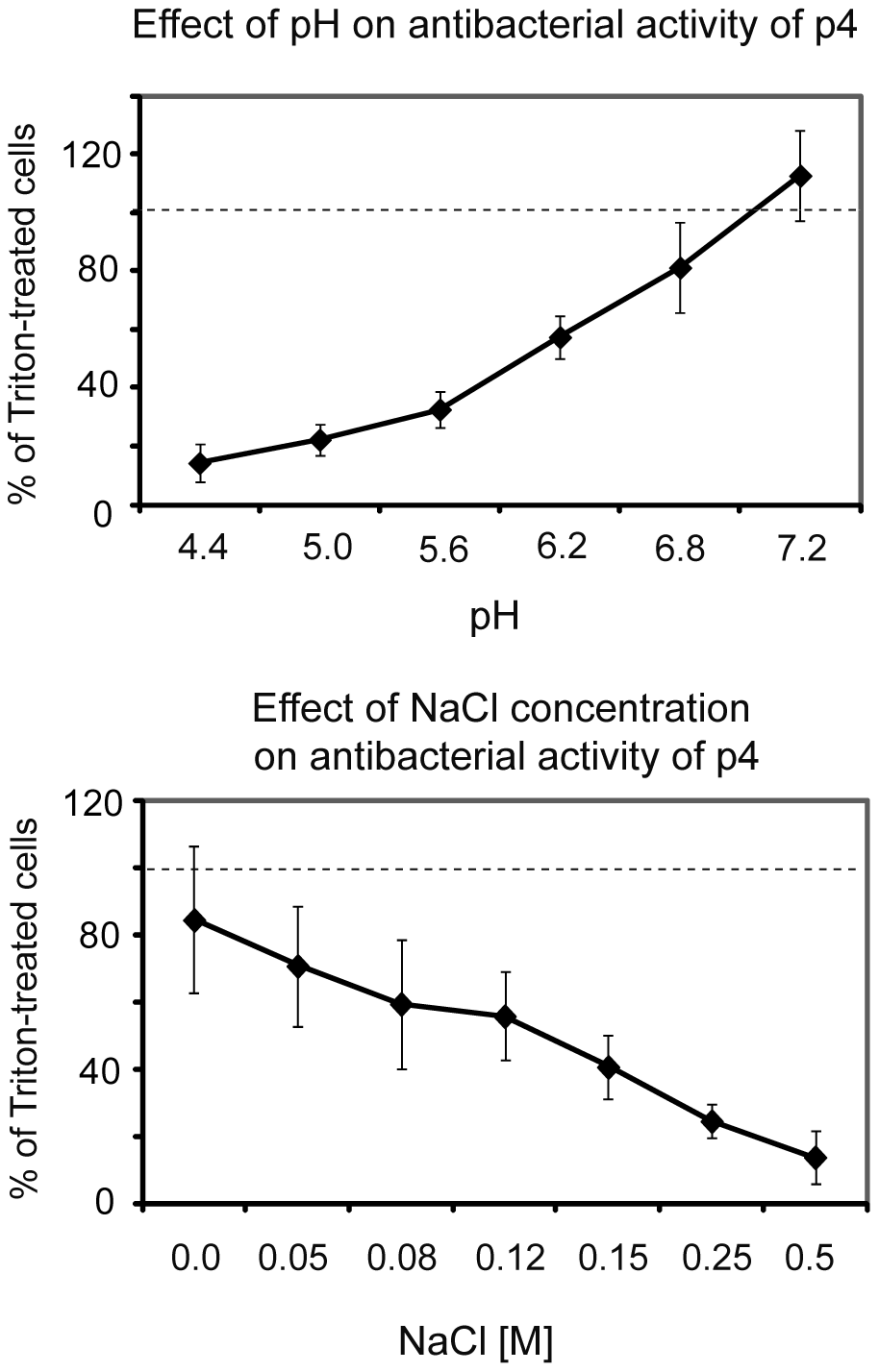
Chemerin peptide 4 exhibits pH- and salt-dependent lytic activity against *E.coli*. Chemically synthesized peptide 4 was tested against *E. coli* JM83 using cytoplasmic beta-galactosidase release assay. Bacteria were incubated with 10 µM peptide 4 for 0.5 h. Maximum (100%) lysis was set by the beta-galactosidase activity present in supernatants from bacteria treated with 1% Triton ×100. The results are expressed as the mean ± SD of three independent experiments.

Like other potent anti-microbial peptides, we hypothesize that the highly positively- charged p4 (Table 2) interacts with negatively-charged bacterial surfaces to disrupt membrane integrity. To ask if p4 causes direct bacterial lysis, we used a β-galactosidase reporter *E. coli* strain, where cytoplasmic β-galactosidase is released into the supernatant following effective lysis [27]. Indeed, incubation with 10 µM of p4 released β-galactosidase suggesting a direct lytic effect. P4 was most active at neutral physiological pH and in low salt, although it seemed to retain activity in physiological (0.15 M) salt concentration (Fig. 5). These data suggest that although charge mostly governs the antimicrobial activity of p4, other mechanisms, such as those based on hydrophobic interaction also play a role in its activity.

## Discussion

At least four notable observations have emerged from our analyses of endogenous skin-derived chemerin and synthetic chemerin peptides as novel anti-microbial agents. First, endogenous chemerin is abundant in human epidermis in situ and well-positioned to provide antimicrobial protection. Second, chemerin-replete exudates from primary skin cultures inhibit bacteria growth and chemerin seems to represent a quantitatively significant fraction of anti-bacterial activity in the products of cultured keratinocytes. Third, the highly positively-charged chemerin domain Val^66^-Pro^85^ embodies the majority of the anti-microbial activity, which is comparable in potency to other antimicrobial proteins. Finally, Val^66^-Pro^85^ likely functions by direct bacterial lysis.

Antimicrobial (poly)peptides may either act as a intact molecules or smaller peptide derivatives [28]. Using recombinant chemerin (pro-chemS163, chemS157 and chemR125) we previously demonstrated that the inhibitory C-terminal peptide present in chemerin holoprotein, pro-chemS163, must be removed for full antibacterial effects [13]. In this work we show that chemerin fragment Val^66^-Pro^85^ (p4) recapitulates the activity of longer chemerin isoforms such as chemS157, which are already devoid of the inhibitory C-term peptide. These data suggest that Val^66^-Pro^85^ domain is largely responsible for the antimicrobial activity of chemerin, and that all chemerin isoforms containing this domain will likely be equipped with antimicrobial potential, provided that they lack the inhibitory C-terminal fragment. Consistent with this, both chemS157 and chemR125 isoforms have similar antimicrobial activity against *E. coli*, and both contain the Val^66^-Pro^85^ fragment [13]. Although it remains to be determined which chemerin isoform(s) are present in epidermis, the proteolytic microenvironment present in pathogen-challenged epithelium will likely be sufficient to activate the antibacterial activity of chemerin. Pro-chemS163 might be converted to an active “antimicrobial” form(s) by proteinases produced by epithelial cells. These may include kallikreins [29]. Alternatively, the epithelium-colonizing pathogens that use proteinases as virulence factors might provide another source of proteinases capable of converting pro-chem163 to forms equipped with bactericidal activity, e.g. forms that lack the inhibitory C-term but contain Val^66^-Pro^85^ domain, such as StpB secreted by *S. aureus* [14].

Elevated plasma chemerin levels have been reported in patients with metabolic syndrome [30]. In our preliminary study, there was no correlation with elevated skin levels of chemerin and the patients' BMI (Table 1).

Previous studies used C-terminal chemerin peptides to characterize sequence determinants required for chemerin bioactivity. Based on these studies, the critical role of F^149^-S^157^ in mediating chemotactic activity through CMKLR1 receptor was demonstrated [31]. Using a similar experimental strategy, we here identified Val^66^-Pro^85^ as a specific chemerin domain responsible for its anti-microbial activity. Collectively, these data provide new evidence that the chemotactic and anti-bacterial activity are associated with the different chemerin region(s).

The major antimicrobial peptides in human epidermis are synthesized by keratinocytes in the stratum granulosum and are delivered into the outer skin layer-stratum corneum, where they contribute to maintaining a barrier against microbial assault [32]. The localization of chemerin in lower layers of the skin together with spectrum of targeted microorganisms suggest a protective function of chemerin following skin disruption, in *E. coli* or *Candida*-infected burn or surgical wounds, for example.Val^66^-Pro^85^ was effective against several microbial species known to cause or worsen skin conditions. However, it shows some selectivity since it exerted the strongest antimicrobial effect against *E. coli* and *C. albicans*. Although the antimicrobial activity of peptide 4 was apparent over a broad pH range and salt concentration, it was the most effective under low salt and neutral pH conditions. Healthy skin surface is well-known to be acidic. However, it also shows an increasing pH-gradient from the surface of the uppermost skin layer-stratum corneum to the deeper layer-stratum granulosum (pH 5 to pH 7.4), [33], [34]. Likewise, the salt concentration at the skin surface is known to vary and depend on sweat which contains approximately 40 mM salt (or more once the skin becomes dry) [35]. Therefore, chemerin peptides are likely to be fully active in skin environment.

Several studies including ours demonstrated that chemerin may contribute to skin defense after proteolytic cleavage through recruitment of pDCs [6], [7], [10], [11], [12], [29]. In addition, results reported in this work suggest that chemerin serves as antibacterial agent in epidermis. Therefore it appears that the biological activities of proteolytically-processed chemerin and its role in skin are much more complex that was originally proposed, since chemerin may operate at multiple levels in skin defense. Local regulation of chemerin expression, and/or activation of pro-chemS163 by proteolytic cleavage, may represent a novel mechanism regulating epithelial cell resistance to bacterial damage. Pathogen-induced weakening of epithelial integrity, and disruption of the antimicrobial defense system of the epithelial layers, would both have profound consequences for development of pathogenic conditions. Therefore, a better understanding of the mechanisms underlying the protective abilities of epithelial cells against pathogens may provide ways to intervene in skin diseases. Manipulation of chemerin levels and bioactivity or the use of chemerin-derived peptides may be a novel therapeutic approach to treat skin infections.
