# Correction: Chemerin Is an Antimicrobial Agent in Human Epidermis

**DOI:** 10.1371/annotation/4dfd522c-f0fd-40db-aadc-44cbef367a40

**Published:** 2013-10-23

**Authors:** Magdalena Banas, Katarzyna Zabieglo, Gopinath Kasetty, Monika Kapinska-Mrowiecka, Julia Borowczyk, Justyna Drukala, Krzysztof Murzyn, Brian A. Zabel, Eugene C. Butcher, Jens M. Schroeder, Artur Schmidtchen, Joanna Cichy

An organization providing funding the grant to the last author (JC) were incorrectly omitted, and a funding organization for that grant was also incorrectly given in the Funding Statement. The first sentence of the Funding Statement should read: “This work was supported in part by the grant from the Foundation for Polish Science TEAM/2010-5/1, co-financed by the European Union within European Regional Development Fund and Polish National Science Center grant 2011/02/A/NZ5/00337 (to JC)." 

